# Oral favipiravir for patients with delayed SARS-CoV-2 viral RNA clearance: a case series

**DOI:** 10.1186/s13054-020-03288-5

**Published:** 2020-09-25

**Authors:** Dian Fu, Ruiyuan Cao, Lei Zhao, Wei Li, Wu Zhong, Jiqiu Wen

**Affiliations:** 1 National Clinical and Research Center of Kidney Diseases, Jinling Hospital, Nanjing University School of Medicine, Nanjing, 210002 China; 2 Wuhan Huoshenshan Hospital, Wuhan, 430100 China; 3 National Engineering Research Center for the Emergency Drug, Beijing Institute of Pharmacology and Toxicology, Beijing, 100850 China

**Keywords:** Favipiravir, SARS-CoV-2, COVID-19, viral RNA persistence

Dear Editor,

The COVID-19 pandemic has caused 14,893,706 infections and 613,879 deaths in 188 countries worldwide as of July 22, 2020, posing the largest world health crisis [1]. Although the pandemic is well controlled in China and the incidence is currently very low, we have observed a number of patients with delayed SARS-CoV-2 viral RNA clearance in the upper respiratory tract (more than 30 days), which combined with asymptomatic carriers, limits the prospect of eliminating the disease. The emergence of patients with delayed viral RNA clearance and healthy viral carriers is a major concern, not only in terms of disease control, but also due to the possible long-term damage to the patients’ health. Previously, we have reported that favipiravir, a broad-spectrum antiviral drug approved in Japan and China, potently inhibits SARS-CoV-2 with a 50% effective concentration (EC_50_) of 61.88 μM in vitro, indicating its antiviral potential [2]. Notably, favipiravir significantly reduced the time to viral clearance in an open-label nonrandomized controlled trial [3]. Here, we report a series of patients with considerably delayed SARS-CoV-2 RNA clearance and the treatment efficacy of favipiravir in this population.

From March 26, 2020, we administered oral favipiravir (two doses of 1600 mg on day 1 and 600 mg twice per day on days 2–10 or until SARS-CoV-2 RNA negative) to nine asymptomatic rehabilitation patients. Eight patients were analyzed, and one patient was lost to follow-up due to transfer to another hospital. Of the eight patients included in this analysis, one received the full 10-day course of favipiravir, and seven received 4 to 9 days of favipiravir treatment.

The demographic and clinical characteristics of the eight patients are shown in Table 1. The median duration of positive detection of SARS-CoV-2 viral RNA in patients before the initiation of favipiravir treatment was 61.0 days (interquartile range, 52.8 to 67.3 days). Coexisting conditions included hypertension (four patients), diabetes (two patients), coronary heart disease (one patient), and malignant tumor (two patients). No interruption of treatment occurred due to adverse reactions.

**Table 1:** Demographic and clinical characteristics of patients

Characteristic	Patients (***N*** = 8)
Median age (IQR), years	60.5 (47.5–68.5)
Male sex, no. (%)	4 (50.0)
Oxygen support, no. (%)	0
Body mass index (kg/m^2^), median (IQR)	22.2 (20.0–26.8)
Median duration of positive RNA detection before favipiravir therapy (days), median (IQR)	61.0 (52.8–67.3)
Other therapies before favipiravir therapy, no. (%)	
Chloroquine phosphate	4 (50.0)
Umifenovir	3 (37.5)
Entecavir	1 (12.5)
Lianhua Qingwen granules	2 (25.0)
Thymopeptides	4 (50.0)
Pidotimod	4 (50.0)
Plasma transfusion	2 (25.0)
Coexisting conditions, no. (%)	
Any condition	6 (75.0)
Hypertension	4 (50.0)
Diabetes	2 (25.0)
Coronary heart disease	1 (12.5)
Malignant tumor	2 (25.0)
Immunoglobulin level (AU/mL), median (IQR)	
IgM	12.5 (5.1–25.2)
IgG	102.7 (56.1–162.5)

*IgG* immunoglobulin G, *IgM* immunoglobulin M, *IQR* interquartile range

Over the 14-day follow-up period, the median duration of viral shedding was 3 days (interquartile range, 2 to 6 days) and one patient remained SARS-CoV-2 RNA-positive after 14 days (Fig. 1a). Notably, seven of eight patients showed a rapid viral clearance within 6 days. One patient kept sustained positive detection of SARS-CoV-2 viral RNA in the upper respiratory tract during the 14-day follow-up (Fig. 1b). The persistence of viral RNA detection in individual patients is shown in Fig. 1c. Seven patients were discharged after two consecutive negative viral RNA tests performed at least 24 h apart. The patients were followed for about 1–2 months for the detection of viral nuclear acid in the throat swabs, and the patients remained negative (Fig. 1d).

**Fig. 1:**
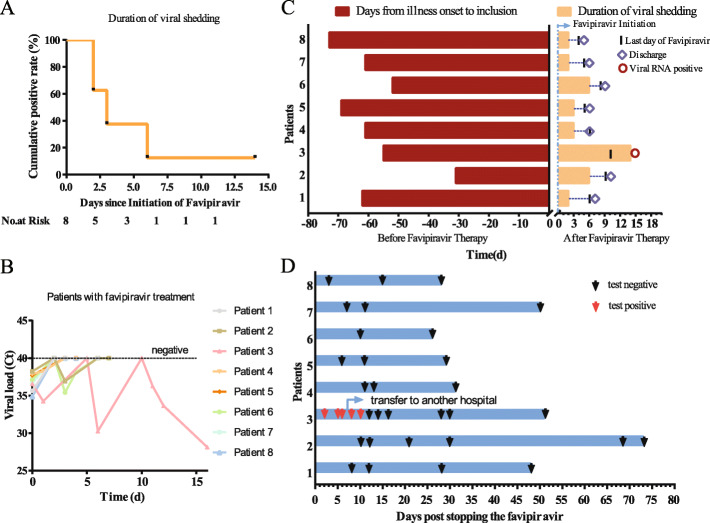
The status of SARS-CoV-2 viral RNA detection in patients. **a** The Kaplan-Meier estimates of the duration of SARS-CoV-2 RNA detection after starting favipiravir. **b** The change from baseline of SARS-CoV-2 viral RNA load by quantitative real-time RT-PCR. **c** The detection of SARS-CoV-2 viral RNA in individual patients. Day 0 is the day on which treatment with favipiravir was initiated. For each patient, the red bar shows the duration of positive SARS-CoV-2 RNA in throat swabs from illness onset to inclusion, and the yellow bar shows the duration of viral shedding after starting favipiravir. The vertical line segment and diamond represent the last day of favipiravir treatment and the day of discharge, respectively. The red circle indicates that viral RNA was detected in the patient’s swab at the end of the 14-day follow-up period. **d** Follow-up results after favipiravir stopped

It has been reported that the persistence of intestinal SARS-CoV-2 shedding in some patients has led to their re-admission after their pneumonia had resolved [4]. Antiviral therapies appear to be an important means to resolve the problem of viral persistence. Our study suggests that favipiravir is worth further investigation as a common and widely used method of treating asymptomatic convalescent patients and carriers. The small size of this cohort and the relatively short period of follow-up limit the strength of evidence obtained by this study, and the results should be interpreted with caution. However, the rapid elimination of viral RNA in seven of the eight patients strongly suggests that administration of favipiravir may have played a role in terminating the viral RNA persistence. Randomized controlled trials are required to determine the efficacy of favipiravir for terminating SARS-CoV-2 shedding in convalescent patients and healthy carriers with delayed viral clearance.

## Data Availability

The datasets generated and/or analyzed during the current study are available from the corresponding authors on request.

## Abbreviations

COVID-19: Coronavirus disease 2019
SARS-CoV-2: Severe acute respiratory syndrome coronavirus 2
